# Antenatal maternal hypoxia: criterion for fetal growth restriction in rodents

**DOI:** 10.3389/fphys.2015.00176

**Published:** 2015-06-08

**Authors:** Eeun Amy Jang, Lawrence D. Longo, Ravi Goyal

**Affiliations:** ^1^Department of Basic Sciences, Center for Perinatal Biology, School of Medicine, Loma Linda UniversityLoma Linda, CA, USA; ^2^Epigenuity LLCLoma Linda, CA, USA

**Keywords:** intra uterine growth restriction, intra uterine growth retardation (IUGR), small for gestational age, low birth weight, maternal stress, developmental origins of adult health and diseases, fetal origins hypothesis, fetal programming

## Abstract

Rodents are a useful model for life science research. Accumulating evidence suggests that the offspring of mice and rats suffer from similar disorders as humans when exposed to hypoxia during pregnancy. Importantly, with antenatal hypoxic exposure, human neonates demonstrate low birth weight or growth restriction. Similarly, with antenatal hypoxic exposure rodents also demonstrate the fetal growth restriction (FGR). Surprisingly, there is no consensus on the minimum duration or degree of hypoxic exposure required to cause FGR in rodents. Thus, we have reviewed the available literature in an attempt to answer these questions. Based on studies in rats, birth weight reduction of 31% corresponded to 10th percentile reduction in birth weight curve. With the similar criterion (10th percentile), in mice 3 days or more and in rats 7 days or more of 14% or lower hypoxia administration was required to produce statistically significant FGR.

## Introduction

Antenatal stressors during pregnancy can lead to fetal diseases as well as the programing of disorders that occur in adulthood (Barker, 2002; Goyal and Longo, 2013; Goyal et al., 2013). Hypoxic stress in pregnancy is common and occurs in various scenarios such as at high altitude, maternal smoking, congestive heart failure, heart valvular diseases, pulmonary diseases, acute/chronic respiratory tract infections, anemia, preeclampsia, placental insufficiency, and others. When a fetus experiences hypoxic stress it redistributes its cardiac output to preferentially perfuse the heart and brain at the expense of other organs (Peeters et al., 1979; Kitanaka et al., 1989; Longo et al., 1993). This relative hypoxia can lead to various disruptions in normal fetal development and eventually to disorders in postnatal life. Antenatal maternal hypoxia has been shown to cause altered cardiac growth and neonatal vascular function, permanent neurological deficits, pulmonary arterial dysfunction, atherosclerosis, and fetal growth restriction (FGR) (McCullough et al., 1977; Unger et al., 1988).

The birth weight of an infant is an important factor to predict its chances of survival, healthy growth, and development. According to the Center for Disease Control (www.cdc.gov) low birth weight (LBW) has been defined as a birth weight of less than 2500 g. LBW could be a result of genetic background without any obvious factor inducing it. In such cases, the LBW infant is referred to as small for gestational age (SGA). However, placental, fetal, or maternal factors may be responsible for LBW. In such cases, in which the fetus is unable achieve its full genetic potential as a consequence of some known factor is diagnosed as FGR. FGR is one of the major complications of antenatal hypoxic exposure in humans, and defined as fetal weight below the 10th percentile of the population for its gestational age resulting from a pathological cause. Of note, FGR affects 7–10% of pregnancies or about 20.5 million infants each year (de Onis et al., 1998; Reece, 2008). FGR increases not only neonatal mortality and morbidity but the risk of diseases during adulthood as well (Hanson and Gluckman, 2008). Neonatal complications of FGR include: aspiration of meconium, neonatal hypoxia, hypoglycemia, and neonatal ischemic encephalopathy. The long-term sequelae during adulthood include lower IQ, increased risk of hypertension, metabolic syndrome, and cardiovascular diseases. The increased risk of diseases in adulthood aligns well with the developmental origins of adult health and disease (DOHaD) hypothesis. Considerable evidence demonstrates a correlation between LBW and subsequent development of cardiovascular diseases (Barker, 1992; Eriksson et al., 2002, 2007). Our previous studies have confirmed that LBW in mice may cause dysregulation of glucose metabolism as well as hypertension in later life with dysregulation of the renin-angiotensin system (Goyal and Longo, 2013; Goyal et al., 2013).

Because of these implications and far-reaching consequences, there is considerable interest in using rodent models in the study of hypoxia-induced FGR. While rodents have been used for more than two decades for this purpose, to date we know of no systematic review to determine what concentrations, durations, and methods of administering hypoxia lead to FGR. Thus, the aim of this report is to determine the parameters of hypoxia that induce FGR in the rodent model. The major questions we asked were: What duration of hypoxia administered will lead to FGR offspring in rodents? When is the optimal time during gestation to administer hypoxia to cause FGR in rodent offspring? What concentration of oxygen administered will lead to FGR offspring in rodents? What is the effect of hypoxia on litter size and maternal food intake? What standards should investigators use to define FGR in the rodent offspring?

## Methods

We conducted a PubMed search using combinations of different study terms such as FGR, fetal growth retardation, intra-uterine growth restriction, intra-uterine growth retardation, LBW, small for gestation age as the first keyword, mice or rats as a second keyword, and hypoxia as a third keyword. Our search included all studies indexed in Pubmed. From these searches, we identified six studies in the mouse or 22 in the rats that were relevant to antenatal maternal hypoxia-induced FGR. Multiple studies from the same investigators using the same hypoxia-induced FGR model were included if they reported different parameters or difference in % of weight reduction. A host of studies deemed irrelevant to the current research questions were excluded. These included studies using methods such as uterine artery ligation, uterine carunculectomy, and studies examining other hypoxia-induced variables such as birth defects, gene expression, and review articles. In two studies (Ravishankar et al., 2007; Dolinsky et al., 2011), pup birth weights were not reported and were excluded from analysis. Also, one of these studies administered both hypoxia and a nitric oxide inhibitor to the pregnant dam with a significant reduction in pups birth weight (Ravishankar et al., 2007). Another study that used hypoxia and uterine artery ligation to induce FGR was also excluded (Thordstein and Hedner, 1992).

Of further consideration, investigators reported their findings of FGR in a variety of manners. Some reported the average weights of both normoxic and hypoxic groups, with the standard deviation and *p*-value to indicate significance. Others reported a percentage difference in weight with *p*-value. To compare the results from all studies, whenever possible, we converted all results to weight difference in percentage.

## Results and discussion

### What duration of hypoxia administered will lead to FGR offspring in rodents?

Quite obviously, this question is complicated as being a function of both oxygen concentration and the duration of administration. In the case of the rat (Table 1), most studies administered hypoxia for 5–7 days during the last week of pregnancy. The longest duration of hypoxia administration was from day post conceptional (DPC) 7–21 (14 days) with 10% oxygen. This resulted in birth weight reduction by 16.9% (*p* < 0.01) (Wang et al., 2009). In comparison, the shortest duration of hypoxia to rat dams, was the exposure to 10% oxygen for 3 h on DPC 20 without development of FGR (Huang et al., 2004). Another study administered 9.5–10.5% oxygen for either 3 h, 1 day, or 11 days to three different groups of rats before sacrificing the mother on DPC 20 (Huang et al., 2004). Fetal weight in the 3 h and 1 day groups showed no significant change from normoxic controls. In contrast, following 11 days exposure, fetal weight was reduced by 31.3% (*P* < 0.05). Both litter size and placental weight were also significantly reduced. Furthermore, in a recent study, 12–14% of oxygen for 7 days (from gestational age 14–21 days) lead to 31% (*P* < 0.050) reduction in pups birth weight (Deng et al., 2015). The shortest duration of hypoxia that resulted in significantly reduced weight in the rat offspring was 3 days. Two studies demonstrated a 20% reduction in fetal weight and 11% reduction in placental weight (*P* < 0.003) with 12 and 14% oxygen administration on DPC 18–21 (Thaete et al., 1997, 2004). Thus, it appears that a minimum duration of 3 days of hypoxic exposure is necessary for inducing FGR.

**Table 1:** **Studies examining hypoxia-induced growth restriction in rats**.

**References**	**Schedule of hypoxia**	**Duration of hypoxia (days)**	**% O_2_**	**% Reduced in weight**	***P*-value <**	**Litter size**	**Food intake**
Huang et al., 2004	DPC20	3 h	9.5–10.5	NS	NS	NS	NR
Huang et al., 2004	DPC19 → 20	1	9.5–10.5	NS	NS	NS	NR
Thaete et al., 2004	DPC18 → 21	3	12	10.2	0.001	NS	NR
Thaete et al., 1997	DPC18 → 21	3	14	20	0.003	NS	NR
Bahtiyar et al., 2007	DPC17 → 21	4	10	4.9	0.02	NR	NR
Bahtiyar et al., 2007	DPC17 → 21	4	14	NS	NS	NR	NR
Lueder et al., 1995	DPC15 → 20	5	10	8.3	0.05	NS	NS
Jakoubek et al., 2008	DPC14 → 20	6	10	20.1	0.0001	NR	NR
Rueda-Clausen et al., 2009	DPC15 → 21	6	11.5	8.5	0.01	NS	NR
Morton et al., 2011	DPC15 → 21	6	12	Below 15th percentile		NS	NR
Hemmings et al., 2005	DPC15 → 21	6	12	Only male group significant	0.01	NR	NR
Morton et al., 2010	DPC15 → 21	6	12	Significant	0.01	NS	
Xu et al., 2006	DPC15 → 21	6	12	12.5	0.05	NR	Reduced
Williams et al., 2005a	DPC15 → 21	6	12	10	0.001	NR	Reduced
Williams et al., 2005b	DPC15 → 22	6	12	10	0.001	Reduced	Reduced
Reyes et al., 2015	DPC15 → 21	6	11	F–12%; M–14%		NR	Reduced
Tapanainen et al., 1994	DPC14 → 21	7	13–14	24	0.0001	NS	NR
Deng et al., 2015	DPC14 → 21	7	12–14	31	0.05	NR	NR
Huang et al., 2004	DPC9 → 20	11	9.5–10.5	31.25	0.05	Reduced	Reduced
Van Geijn et al., 1980	DPC10 → 22	12	9.5	36	0.001	61% Decrease	NR
Vosatka et al., 1998	DPC9 → 21	12	10	30	0.0001	NR	NS
Wang et al., 2009	DPC7 → 21	14	10	16.9	0.01	NS	NR

*DPC, day post coitum; F, females; M, Males; NR, Not reported; NS, no significant difference*.

In contrast to the rat, the mouse had less variation in duration of hypoxia (Table 2). In these studies, the longest hypoxic duration administered was 8 days (Rueda-Clausen et al., 2014), while the shortest was 12 h. In a recent study, 8 days exposure of 10.5% hypoxia caused 36% decrease in pups birth weight as compared to normoxic controls (Rueda-Clausen et al., 2014). Moreover, increased neonatal mortality was also reported in this study without any decrease in litter size. Two groups, Gortner et al. (2005) and Tomlinson et al. (Tracy et al., 2010), administered 3 days of hypoxia in the last week of pregnancy, at 10 and 12% oxygen, respectively. Gortner et al. reported a 28.9% decrease in pups weight, while Tomlinson only mentioned a statistically significant decrease in pup weight with *P* < 0.05. Our studies with 10.5% oxygen for 48 h between DPC 15.5–17.5 demonstrated no significant change in pups birth weight (Gheorghe et al., 2010; Goyal et al., 2011a,b). In another study, hypoxia was given for 12 or 24 h on DPC 11.5–12.5, at 8% oxygen concentration (Ream et al., 2008). Importantly 8% oxygen is a comparatively more severe hypoxic exposure, than that of other studies, which used 10–12% oxygen. Additionally, this study is unique from other reports in that it used a low oxygen concentration and administered hypoxia to the pregnant mouse during the second week post coitum. While the 12-h hypoxia group showed no significant difference in fetal weight from the controls, the 24-h group showed a 26% reduction in fetal weight compared to the control group. This is in contrast to our findings that 48 h of hypoxia showed no significant change in birth weight. However, in the study by Ream et al., the investigators used 8% hypoxia, and in our studies we used 10.5% hypoxia. Also, in other studies we observed that pre-conceptional 10% hypoxia for 4 days failed to induce FGR in mice (unpublished observation). Thus, based on the studies on rodents, we conclude that 3 days of moderate hypoxia in the 10–14% range results in significant FGR. Also, increasing the severity to 8% for at least 24 h can produce a significant decrease in birth weight. Notably, 8% oxygen in the rabbit is a critical level associated with fetal death (Power et al., 1977).

**Table 2:** **Studies examining hypoxia-induced growth restriction in mice**.

**References**	**Schedule of hypoxia**	**Duration of hypoxia (days)**	**% O_2_**	**% Reduced in weight**	***P*-value <**	**Litter size**	**Food intake**
Ream et al., 2008	DPC11.5 → 12.5	12 h	8	NS	NS	NR	NR
Ream et al., 2008	DPC11.5 → 12.5	1	8	26	0.05	NR	Reduced
Goyal et al., 2011a	DPC15.5 → 17.5	2	10.5	NS	NS	NS	NR
Gortner et al., 2005	DPC14 → 17.5	3	10	28.9	0.001	NS	NR
Tracy et al., 2010	DPC15.5 → 18.5	3	12	Significant	0.05	NS	Reduced
Rueda-Clausen et al., 2014	DPC10.5 → 18.5	8	10.5	36	0.05	Reduced	NR

*DPC, day post coitum; F, females; M, Males; NR, Not reported; NS, no significant difference*.

### What is the effect of hypoxia on litter size and maternal food intake?

Most of the studies did not report the effect of hypoxia on litter size. A study which administered hypoxia for 11 days reported reduced litter size, but failed to mention the exact number (Huang et al., 2004). Another study with severe hypoxia exposure of 9.5% for 12 days demonstrated significant reduction in litter size (Van Geijn et al., 1980). Both these studies administered the hypoxic insult for more than half of the gestation period. However, studies for 6 days of hypoxia of 12% oxygen showed no significant difference in the litter size in same rat strain (Rueda-Clausen et al., 2009, 2011; Morton et al., 2010). Similarly, 5 days hypoxia (E15–20) of 10% oxygen did not lead to significant change in litter size (Lueder et al., 1995). In the same study no effect of hypoxia was reported on the maternal food intake. Similarly, no effect of hypoxic exposure on maternal food intake was reported in response to 12 days hypoxic exposure (10% from E9 to E21) (Vosatka et al., 1998). In contrast, few other groups (Tables 1, 2) have reported reduced maternal food intake in response to hypoxia (Huang et al., 2004; Williams et al., 2005a,b; Ream et al., 2008; Tracy et al., 2010; Reyes et al., 2015). However, in other studies from the same group there is no mention of changes in maternal food intake in response to hypoxia exposure (Rueda-Clausen et al., 2009, 2011, 2014; Morton et al., 2010). Therefore, further investigations are needed to clarify how hypoxia-influences litter size and maternal food intake.

### When is the optimal time during gestation to administer hypoxia to cause FGR in rodent offspring?

In reviewing these reports, we noted that most studies examined the effects of hypoxia during the last week of pregnancy. Only a handful of studies examined the effects of hypoxia during the second week, and no studies examined these effects during the first week. The question is, to what extent does the hypoxic exposure during different time periods of gestation affect offspring birth weight?

In the mouse three of the six studies administered hypoxia in the third week of pregnancy, while three studies administered hypoxia in the second week. The three studies administering hypoxia in the last week reported FGR with a statistically significant difference in the pups' birth weight (Gortner et al., 2005; Tracy et al., 2010; Rueda-Clausen et al., 2014). Gortner et al. reported a ~29% reduction of birth weight in the hypoxic group (Ream et al., 2008). In comparison, administering hypoxia in the second week resulted in a 26% reduction of birth weight.

In the rats, 18 of the 22 studies administered hypoxia in the last week of pregnancy, while 4 of the 22 studies administered hypoxia in the second and third weeks (Table 1). Again, none of the studies administered hypoxia in the first week. These reports suggest that irrespective of second or last week, the development of FGR is more dependent upon both duration and degree of the hypoxic insult. Importantly, in humans and most other species, most fetal growth occurs during the last trimester of pregnancy, and some would argue that this is the most critical period to examine the effect of antenatal maternal hypoxia on newborn birth weight.

### What concentration of oxygen administered will lead to FGR offspring in rodents?

To answer this question, we looked at percentage of oxygen administered in each study and the effect produced in pup weight. The percentage of oxygen administered varied from 8 to 12% in the mice studies and 9.5–14% in the rat studies. Significant reductions in pup weight were reported at each of these oxygen concentrations. Concurrently, non-significant reductions in pup weight were reported at many levels of oxygen as well, including the two ends of the spectrum at 8 and 14% oxygen (Huang et al., 2004; Bahtiyar et al., 2007; Tracy et al., 2010; Goyal et al., 2011a,b). This variability was dependent on the duration of hypoxia given in each study. While, oxygen levels below 11% produced significant reduction in pup weight (Van Geijn et al., 1980; Lueder et al., 1995; Vosatka et al., 1998; Huang et al., 2004; Thaete et al., 2004; Gortner et al., 2005; Bahtiyar et al., 2007; Jakoubek et al., 2008; Ream et al., 2008; Wang et al., 2009), two studies that administered hypoxia (8 to 9.5% oxygen levels) for 1 day or less did not achieve significant FGR (Huang et al., 2004; Ream et al., 2008). In rats, Huang et al. administered 9.5–10.5% oxygen for 3 h as well as for a day and reported no significant reduction in pup birth weight. Ream et al. administered 8% oxygen for 12 h to mice and reported pup weight reduction that was not significant (Ream et al., 2008). Thus, the duration of hypoxic exposure appears to be a major factor in determining the development of FGR than the degree of hypoxia *per se*.

### What standards should investigators use to define FGR in the offspring?

Lastly, we examined the criteria investigators used to determine a positive finding of FGR in these studies. Each investigator used a statistically significant difference (*P* < 0.05), compared to normoxic control to label the pups as FGR. In more than 100 control litters, a group of investigators further determined how this statistically significant weight translated into percentile, and defined FGR as birth weight below the 15th percentile of sex-matched controls (Rueda-Clausen et al., 2009, 2011; Morton et al., 2010). In a recent study in rats, 31% reduction in birth weight was reported for 10th percentile of birth weight for gestational age reference curve (Deng et al., 2015). In this study, FGR incidence was 54.4% in the hypoxic group as compared to 5.6% FGR in the normoxic group.

As noted above, in human neonates, the weight criterion for FGR is below the 10th percentile of the population at the respective gestational age. Thus, a statistically significant reduction in weight does not translate directly to a weight below the 10th percentile of the population. The average birth weight of a near-term human newborn is about 3200 g. The weight criterion for FGR is below 2500 g. This translates into a minimum of 22% reduction in weight, at which point the perinatal mortality rate is 5–30 times greater than that of a newborn of normal weight. At 1500 g, or a 53% reduction in weight, the perinatal mortality rate increases to 70–100 times greater. In this review, we identified only two studies in mice (Ream et al., 2008; Rueda-Clausen et al., 2014) and one study in the rat (Tapanainen et al., 1994), which mentioned significant increase in newborn pups mortality following maternal hypoxia. All other studies did not report neonatal mortality in response to maternal hypoxia exposure. However, further investigations are needed to evaluate the relationship of rodents pups birth weight and neonatal mortality.

## Conclusion and perspective

We conclude from this analysis that 14% or lower level of oxygen for 3 days or more is sufficient to produce FGR in rodents. Also, the second half of pregnancy (chiefly the third week) is the time frame most studied for hypoxic insult, but earlier exposure may also lead to FGR. Importantly, we propose that in rodents, at least 22% or more reduction in pups birth weight should be considered as FGR.

### Conflict of interest statement

The authors declare that the research was conducted in the absence of any commercial or financial relationships that could be construed as a potential conflict of interest.
